# Impaired experience-dependent maternal care in presynaptic active zone protein CAST-deficient dams

**DOI:** 10.1038/s41598-020-62072-1

**Published:** 2020-03-23

**Authors:** Akari Hagiwara, Naoko Sugiyama, Toshihisa Ohtsuka

**Affiliations:** Department of Biochemistry, Faculty of Medicine, University of Yamanashi, Yamanashi, Japan

**Keywords:** Cellular neuroscience, Motivation

## Abstract

Although sociological studies affirm the importance of parental care in the survival of offspring, maltreatment—including child neglect—remains prevalent in many countries. While child neglect is well known to affect child development, the causes of maternal neglect are poorly understood. Here, we found that female mice with a deletion mutation of CAST (a presynaptic release-machinery protein) showed significantly reduced weaning rate when primiparous and a recovered rate when multiparous. Indeed, when nurturing, primiparous and nulliparous CAST knock out (KO) mice exhibited less crouching time than control mice and moved greater distances. Contrary to expectations, plasma oxytocin (OXT) was not significantly reduced in CAST KO mice even though terminals of magnocellular neurons in the posterior pituitary expressed CAST. We further found that compared with control mice, CAST KO mice drank significantly less water when nurturing and had a greater preference for sucrose during pregnancy. We suggest that deficiency in presynaptic release-machinery protein impairs the facilitation of some maternal behaviours, which can be compensated for by experience and learning.

## Introduction

Parental care is essential for the survival of mammalian offspring and influences their adult lives mentally and physically. Naturally, both parents have responsibilities from nest building to feeding, but because they lactate, the primary responsibility for parental care falls to mothers^1^. As nursing is essential for mammalian survival, the basic neural mechanisms underlying maternal care are likely conserved throughout the mammalian evolutionary tree. Therefore, studying these neural mechanisms in non-human mammalian models such as gene-transgenic mice^2^ can help us understand human maternal care and prevent neglect. In rodents, maternal behaviours such as nest building, gathering pups together in the nest, keeping them warm, and nursing are critical for pup survival.

Complex maternal behaviours require highly motivated and flexible neural systems and have frequently been associated the neuropeptide oxytocin (OXT), which is secreted in both the periphery and the brain^2–5^. Indeed, considerable evidence indicates that OXT promotes maternal behaviour, while OXT deficiency (OXT KO in rats) results in impaired milk ejection without obvious defects in parturition^3^. Similarly, a null mutation of the OXT receptor in mice led to defects in lactation and maternal behaviour^6,7^. Additionally, mice deficient in CD38 (a transmembrane glycoprotein with ADP-ribosyl cyclase activity) also showed defects in maternal behaviour correlating with the reduction of plasma OXT levels^8^. Therefore, OXT and its receptor system are essential for the regulation of maternal behaviour. However, OXT is just one of the many critical players; complex maternal behaviours are also regulated by other neuromodulators including dopamine, serotonin, norepinephrine, and corticotropin-releasing factor, which have been associated with maternal motivation and stress-induced anti-maternal behaviour^2,9–11^. Moreover, a growing number of gene-knockout mouse strains have been reported to exhibit defective maternal behaviour. Indeed, a website search for “abnormal maternal behaviour” retrieved 182 genotypes including OXT receptor KO^6^ and anosmic mice^12^—in which maternal crouching was absent and offspring retrieval was lower than normal—as well as GABA_A_ receptor KO mice^13^ who exhibited abnormal offspring retrieval (MGI, http://www.informatics.jax.org/, as of August 2019).

All maternal behaviours are dictated by neural networks in the brain that are regulated by the release of neurotransmitters and neuromodulators from presynaptic terminals. Presynaptic release is regulated by release-machinery proteins termed cytomatrix at the active zone (CAZ) proteins, including Munc13, RIM, Bassoon, Piccolo, and CAST/ELKS^14,15^. CAST (CAZ-associated structural protein)/ERC2 (also known as ELKS2α) and ELKS/ERC1 (CAST2) are family proteins approximately 120 kDa in size that have coiled-coil regions^16–18^. In the complex comprising CAZ proteins, CAST directly binds to RIM at IWA, a unique COOH-terminal amino acid motif on CAST. Deletion of IWA motif had less effect on CAST localization to the AZ, while deletion of the CAST binding domain (PDZ domain) on RIM resulted in diffuse RIM distribution within the axon, which suggested that CAST acts as an anchor^17^. Previous work has shown that mutant forms of CAST/ELKS homologs in *C. elegans* (ELKS) and *Drosophila* (bruchpilot) exhibit distinct phenotypes, indicating they are required for synapse formation in *C. elegans* and for the promotion of active zone assembly in *Drosophila*^19,20^. Furthermore, analysis of CAST and/or ELKS deficient mice indicated that these proteins affect calcium channel-associated release machinery in retinal photoreceptor neurons, hippocampal neurons, calyx of the Held/MNTB synapses, and pancreatic β cells^21–26^. However, despite the importance of presynaptic events, only a few studies have reported the contribution of presynaptic neurotransmitter release to maternal behaviour^2,27^.

To address this issue, we examined maternal behaviour in CAST KO female mice. We found a significantly lower weaning rate in primiparous CAST KO dams. Our initial analysis of maternal care revealed that reduced crouching time and increased activity were related to the reduced weaning rate. While magnocellular neurons in the posterior pituitary expressed CAST and OXT, OXT release from the pituitary gland was not affected. However, CAST KO dams drank significantly less water than controls and tended to prefer sucrose during pregnancy and nurturing, hinting at reduced motivation to perform maternal behaviour in CAST KO mice. Our finding suggests that ablation of CAST from the neural network impairs neuro-regulation, resulting in abnormal sensitivity during prenatal and postnatal periods.

## Results

### Maternal behaviour was impaired in primiparous CAST KO mice

**Figure 1:**
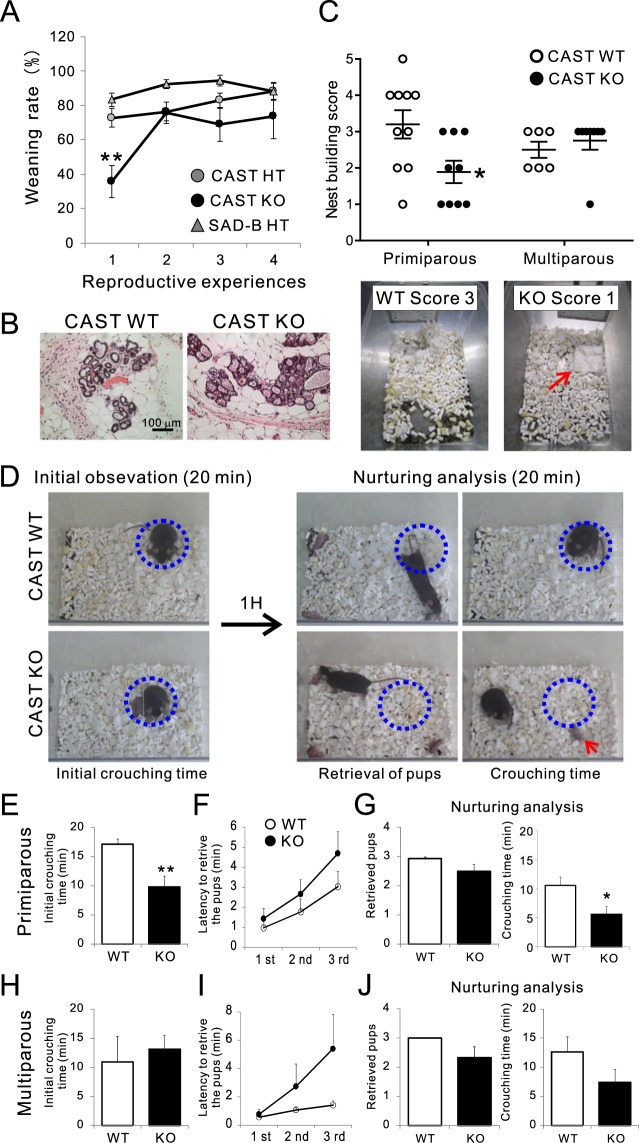
Reproductive performance was impaired in primiparous CAST KO mothers. (**A**) The weaning rate was significantly lower in CAST KO dams than in CAST heterozygous (HT) or other genotype-deficient dams at the first reproductive experience (primiparity); **p < 0.01 by one-way ANOVA, Tukey’s test. **(B)** HE stained sections of the mammary gland. **(C)** Nest-building scores were significantly lower in CAST KO mice at primiparity than in WT mice (WT: 3.20; KO: 1.89; *p < 0.05 by t-test). 5-point nest scoring: 0, untouched nest material; 1, moved nest material; 2, flat nest; 3, <half cupped dome; 4, half-height dome; 5, > half-height or full dome. (bottom) Typical nests of score 3 made by WT mice and score 1 by KO mice. **(D)** Nurturing analysis. Monitoring of WT and KO dams while they took care of newborn pups (P0–3 days). After the 20 min initial observation, pups were deprived of house cage for 1 h. Subsequently, they were placed in each corner of the home cage, except for the nest corner (dotted circle), and then WT or KO dams were returned to the nest and monitored for 20 min (nurturing analysis). **(E–G)** From the video record, the number of pups retrieved (**G**), latency to retrieve each pup (**F**), and crouching time for all three pups (**E**,**G**) were measured. If the number of retrieved pups was less than three, crouching time was 0. The KO mice missed retrieving one of three pups (red arrow in **D**). Crouching time at the initial observation and during nurturing was significantly lower in primiparous KO mice (n = 20) than in WTs (n = 13) **(H–J)** In multiparous KO mice, initial crouching time was similar to that seen in WTs (**H**). While the WT mice retrieved all three pups within 1 min, the KO mice took longer, but the number or pups and the time spent crouching did not differ significantly (**I,J**). WT (3), KO (9); mean ± SEM, *p < 0.05, **p < 0.01 (Student’s *t*-test).

To investigate the role of the presynaptic active zone protein CAST in maternal care, we generated constitutive CAST KO mice by targeting the first coding exon (exon3)^26^. CAST comprises 19 exons and 2 non-coding regions, exon1, and exon2. Immunoblotting demonstrated that full-length CAST (120 kDa) is absent in the KO mice, although the splice isoform (CASTβ) starting from exon6 (about 70 kDa) was detected^22^. Furthermore, database analysis revealed a CAST gene containing spliced 3′ exons that encode a C-terminal splice variant without an IWA motif. Therefore, the CAST gene expresses four principal isoforms, of which the full-length CAST containing an IWA motif is the most common by far^22,24^. The KO mice were viable, but their body weight was slightly smaller than that of WT mice (Male WT = 21.8 g; KO = 18.4 g; *p* < 0.05, Student’s t-test; Female WT = 19.0 g; KO = 16.0 g; *p* < 0.05). Both male and female KO mice were fertile. However, we found that numerous pups with KO dams died during the postnatal period. In fact, weaning rate (pup number at weaning [4 weeks] divided by pup number at birth) was significantly lower in primiparous KO mice than in CAST heterozygous (HT) and SAD-B HT females^28^, which had a typical 70–80% weaning rate (Fig. 1A). Intriguingly, the CAST KO weaning rate recovered to 70% after birthing their second litter.

Although we first suspected a lactation defect was responsible for the reduced weaning rate, histological analysis of mammary glands from primiparous WT and KO mothers revealed no functional abnormalities (Fig. 1B). Next, we considered the possibility of behavioural abnormalities. Normal nurturing behaviour in mice is quite stereotyped, including creating a nest, bringing pups to the nest, and cleaning and crouching over them for warmth and nursing. We found that nest building was impaired in CAST KO mice; the nestlets were left intact by almost half the KO mice (Fig. 1C). Consistent with the recovered weaning rate, nest building scores were improved in multiparous KO mice (Fig. 1C). Next, we considered the possibility of abnormal maternal behaviour in response to postnatal day 0–3 pups. We first observed the behaviour of newly postpartum primiparous WT and KO mice for 20 min (Fig. 1D,E; initial). Although WT mice spent the majority of the period crouching over their pups, KO mice spent less time doing so (Fig. 1E). Following this initial observation, pups were separated from their mothers and went without nursing (food) for 1 h. Maternal behaviour was then assessed in response to three of a dam’s own pups, each of which was placed in one of the three corners of the cage away from the nest (Fig. 1D). WT and most KO mice approached the pups immediately and retrieved them to the nest within 5 min. However, 20% (4/20) of the KO mothers failed to bring the pups to the nest during the observation period, and their crouching time was significantly reduced (Fig. 1G). Similar to nest building and nurturing behaviour, the number of pups retrieved on this test by the KO mice improved after multiple pregnancies (Fig. 1H–J). Nurturing analysis showed that both multiparous WT and KO dams displayed slightly greater crouching time than the primiparous dams (Fig. 1J). Although crouching time in multiparous KO dams did not significantly differ from that in WT dams (*p* = 0.1), 33% (3/9) of them still failed to bring the pups to the nest and their crouching times were counted as 0, which led to the overall lower crouching time. From these results, we can say that even though multiparous KO dams showed improved maternal behaviour (i.e., a better weaning ratio), the neural network underlying their maternal behaviour did not seem to be perfectly remodelled.

**Figure 2:**
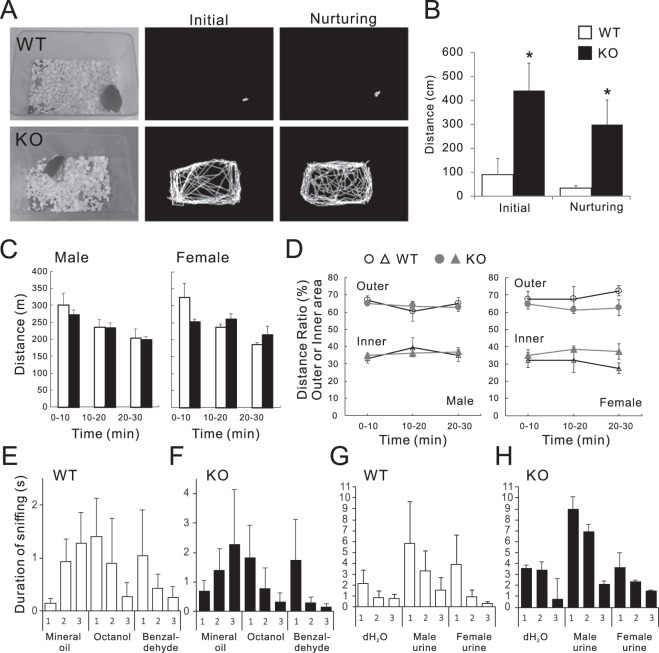
Increased mobility in nurturing CAST KO dams. **(A,B)** Nurturing dams in the home cage were tracked using video capture. The WT mice stayed mostly in the nest for breastfeeding and to keep the pups warm. In contrast, the KO mice moved around the cage. The distance travelled was significantly higher in the KO mice, and correlated with the reduction in crouching time. WT (5), KO (7); mean ± SEM, *p < 0.05 (Student’s *t*-test). **(C,D)** To measure exploratory activity, open field activity was measured with male and virgin female WT and KO mice. The distance travelled by KO mice did not differ significantly from WT males or females. Furthermore, WT and KO mice exhibited a similar trend for traveling mostly in the peripheral zone (outer) compare with the centre zone (inner). Male WT (6), KO (12), Female WT (3), KO (4); mean ± SEM. **(E–H)** WT and KO mice performed an olfactory habituation/discrimination test via a cotton-tip presentation-based task. Six-to-ten-week old mice were pretrained with mineral oil-soaked cotton swabs, then exposed to two different odorants (100 µM octanol and benzaldehyde), with 3 trials per odorant. Sniffing duration indicated that both WT and KO mice were able to detect and discriminate between the distinct odorants (**E,F**). On a separate day, the mice performed a similar task using male and female urine after undergoing pretraining on distilled water. Habituation and discrimination of urine samples was comparable between KO and WT mice (**G,H**). Female WT (7), KO (6); mean ± SEM.

Reduced crouching time in the KO mice did not result solely from retrieval failure, but was also related to abnormal movement during nurturing behaviour (Fig. 2A). Movement tracking revealed a significant increase in the distance travelled around the cage at the initial observation and during nurturing (Fig. 2B). In an open field test, males and virgin females did not differ significantly in distance travelled or preference for centre and peripheral zones (Fig. 2C,D, Table 1). Therefore, the abnormally increased movement was a specific phenotype of nurturing KO females. Because olfactory cues are important for evoking maternal behaviour, we used olfactory habituation and discrimination tests to determine whether virgin WT and KO females could detect and distinguish different odorants (Fig. 2E–H)^29,30^. In these tests, the mouse was first habituated in its cage to the presence of a cotton swab soaked in mineral oil. This pretraining ensured that sniffing was not a response to the novel object. Unlike in a previous report^29^, both WT and KO mice kept a lookout for the cotton swab at the first exposure, and the time spent sniffing increased slightly at the second and third exposures (Fig. 2E,F). Then, the mouse was presented with a cotton swab laced with odours (octanol and benzaldehyde, separately on different trials), and the sniffing duration was recorded. A high duration of sniffing upon each initial exposure, and a decline in subsequent trials for each odorant, indicated the mice detected the novel odorants and habituated to them (Fig. 2E,F). Mice were then tested for their ability to discriminate between male and female urine (Fig. 2G,H). In these cases, because the cotton swab was already known to the mice, sniffing duration for the distilled water-soaked cotton swab was high at the first exposure and decreased slightly on subsequent trials. Similar to the chemical odorant, sniffing duration to the initial male and female urine exposure was high (Fig. 2G,H). From these results, CAST KO mice detected these odorants and discriminated the difference between male and female urine. This test allowed us to determine whether or not the sense of smell in KO mice was normal, but we did not compare sniffing duration between WT and KO mice. This decision was based mostly on sensitivity; sensitivity depends largely on the curiosity and motivation to investigate the odours, which could be affected by the oestrous cycle in the case of the female mice. Therefore, here we indicate that the KO mice had standard olfaction, but further studies should examine whether odorant discrimination using odours of similar chemical structure would be able to detect abnormal olfactory function in KO mice.

**Table 1:** Open field test results.

Male		0–10 min	10–20 min	20–30 min
		WT (n = 6) KO (n = 12)		
Distance (%)	Outer	66.9 ± 2.5 65.0 ± 2.5	60.5 ± 5.7 63.6 ± 5.7	65.0 ± 3.4 62.9 ± 3.4
	Inner	33.1 ± 1.3 35.0 ± 1.3	39.5 ± 2.4 36.3 ± 2.4	34.9 ± 2.4 37.1 ± 2.4
Presence time (seconds)	Outer	435.9 ± 25.6 434.4 ± 12.4	397.6 ± 41.1 413.5 ± 20.4	409.9 ± 36.8 399.9 ± 19.4
	Inner	164.1 ± 25.6 165.6 ± 12.4	202.3 ± 41.1 186.2 ± 20.4	189.9 ± 36.8 199.9 ± 19.4
Resting time (seconds)	Outer	29.87 ± 4.9 41.10 ± 8.1	41.77 ± 9.2 46.17 ± 6.4	51.77 ± 9.3 50.60 ± 6.1
	Inner	10.23 ± 4.0 10.52 ± 1.6	19.60 ± 6.0 18.80 ± 3.9	27.50 ± 12.6 21.73 ± 3.0
	Total	40.10 ± 6.0 51.62 ± 9.0	61.37 ± 9.3 64.97 ± 8.2	79.27 ± 14.0 72.33 ± 6.2
**Female**		**0–10 min**	**10–20 min**	**20–30 min**
		**WT (n = 3)** **KO (n = 4)**		
Distance (%)	Outer	67.7 ± 4.3	67.8 ± 7.2	72.5 ± 3.1
		64.9 ± 3.1	61.4 ± 2.0	62.7 ± 4.6
	Inner	32.3 ± 4.3	32.2 ± 7.2	27.5 ± 3.1
		35.1 ± 3.1	38.6 ± 2.0	37.3 ± 4.6
Presence time (seconds)	Outer	440.03 ± 27.6	421.73 ± 46.3	465.40 ± 24.8
		425.53 ± 26.1	399.98 ± 18.2	381.05 ± 46.0
	Inner	159.97 ± 27.6	178.07 ± 46.3	134.40 ± 24.8
		174.48 ± 26.1	199.83 ± 18.2	218.75 ± 46.0
Resting time (seconds)	Outer	25.13 ± 9.1	41.33 ± 13.1	66.80 ± 15.0
		37.70 ± 3.2	36.25 ± 9.1	45.50 ± 15.3
	Inner	8.33 ± 5.4	17.47 ± 5.1	19.87 ± 11.0
		13.40 ± 4.9	13.50 ± 3.7	28.55 ± 8.5
	Total	33.47 ± 12.0	58.80 ± 14.2	86.67 ± 22.7
		51.10 ± 4.2	49.75 ± 9.4	74.05 ± 17.2

### Triggered maternal behaviour in virgin females was impaired in CAST KO mice

**Figure 3:**
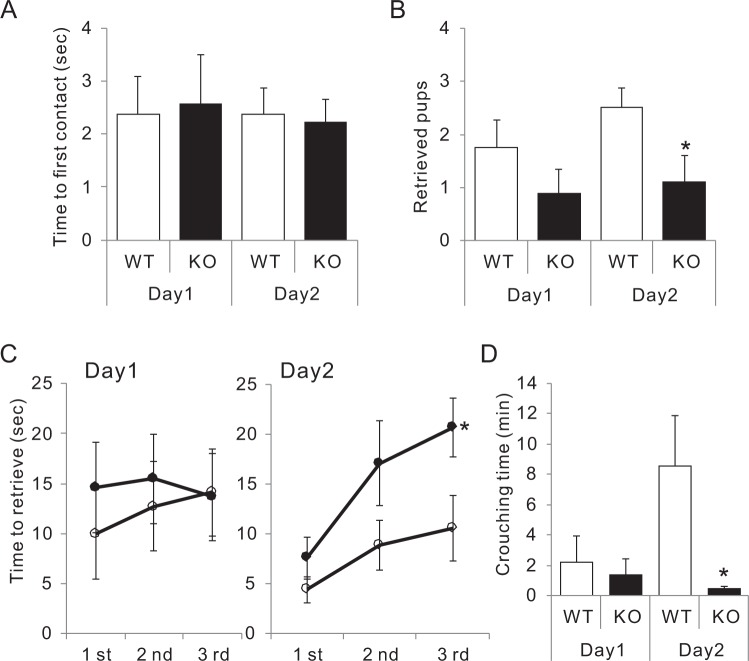
Maternal care in virgin females. **(A–D)** Virgin adult female WT and KO mice were exposed to pups for two consecutive days. The first contact time (**A**), number of retrieved pups (**B**), latency to retrieve pups (**C**), and crouching time over three pups (**D**) were measured. WT (8), KO (9), mean ± SEM, *p < 0.05 (Student’s *t*-test).

Virgin female mice can be induced to engage in maternal behaviour by repeated exposure to newborn pups^31^. Here we examined virgin WT and KO females, and both showed interest in immediately contacting newborn pups (Fig. 3A). However, only 50% of the WT mice (4/8) and 22% of the KO mice (2/9) could successfully retrieve three pups to the nest on the first day, and average crouching time was only a few minutes (Fig. 3). On the following day, the percentage of WT mice that retrieved all pups increased to 75% (6/8), while that for KO mice remained low at 33% (3/9) (Fig. 3B). Furthermore, while 3 WT mice crouched over the pups more than 18 min on the second day of exposure to the pups, none of the KO mice crouched over the pups for more than 1 min (Fig. 3C,D). From these results, we concluded that the neural system that governs switching to maternal behaviour might be inadequate in CAST KO dams.

### CAST KO had little effect on OXT release from the posterior pituitary gland

**Figure 4:**
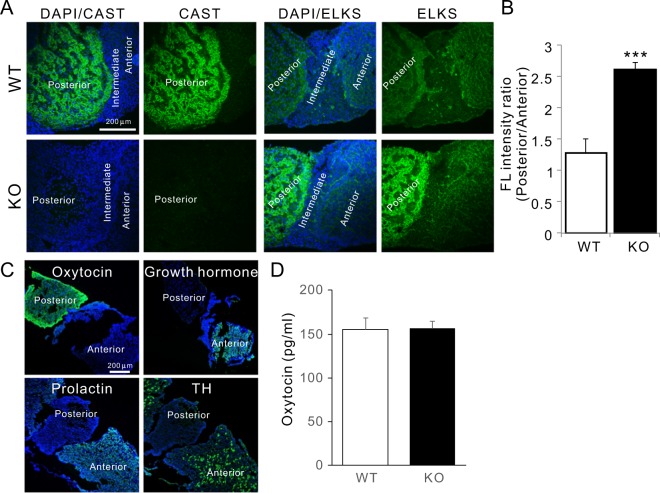
Contribution of OXT release from the pituitary gland. **(A)** Immunohistochemistry showed that CAST was distributed in the posterior pituitary, suggesting the CAST in magnocellular neurons of the hypothalamus might regulate the release of OXT. In KO mice, the expression of family protein ELKS seemed complementarily enhanced in the posterior region. **(B)** Ratio of fluorescent intensity of anti-ELKS antibody between posterior and anterior pituitary indicated the enhancement of ELKS expression in the posterior region of KO mice. WT (4), KO (5), mean ± SEM, ***p < 0.005 (Student’s *t*-test). **(C)** Immunohistochemistry of hormones including OXT, growth hormone, prolactin, and thyroid-stimulating hormone (TH) indicated their distribution in the pituitary gland. Unlike other hormones that were released from the anterior pituitary, OXT was only distributed in the posterior region. **(D)** Concentration of plasma OXT in male WT and KO mice. OXT release to plasma did not differ significantly between WT and KO mice. WT (3), KO (5), mean ± SEM.

Female reproduction and facilitation of maternal behaviour is known to be modulated by oxytocin (OXT), which is synthesized in neurons of the paraventricular and supraoptic nuclei and released to the general circulation from the posterior pituitary and the central nervous system^32^. The pituitary gland is located at the base of brain and comprises an anterior, intermediate, and posterior lobe. While the anterior pituitary contains variety of hormone-releasing cells, the posterior is largely a collection of axonal projections from hypothalamic neurons. Although previous immunohistochemical studies using anti-CAST antibody described the widespread reactivity of CAST in the brain to include the hippocampus, cortex, and retina^17,33^, expression of CAST was not investigated in the pituitary gland. Here, we found CAST expression in the posterior lobe, which was eliminated in the KO mice. This indicated that CAST is expressed in hypothalamic neurons (Fig. 4A). Similar to the retinal examination in the KO mice, in which the family protein ELKS was upregulated^26^, the ubiquitous expression of ELKS in the pituitary glands of the KO mice was specifically enhanced in the posterior lobe (Fig. 4B). Immunohistochemical analysis using anti-hormone antibodies correctly indicated hormone release sites. For instance, OXT was localized to the posterior pituitary and growth hormone, prolactin, and thyroid-stimulating hormone to the anterior pituitary (Fig. 4C). To examine the role of CAST in hormonal release from the posterior pituitary gland, plasma OXT levels were investigated. Peripherally released OXT did not significantly differ between WT and KO mice (Fig. 4D), which was confirmed by normal development of mammary glands in KO dams (for lactation) (Fig. 1B). As expected, other hormones released from the anterior pituitary, including growth hormone and luteinizing hormone, were not significantly different between WT and KO males (Table 2). Therefore, the secretion of OXT from the pituitary might be complemented by upregulation of ELKS (the CAST family protein) in hypothalamic neurons.

**Table 2:** Male released hormone concentrations.

	WT	KO
Growth hormone (ng/ml)	1.99 ± 0.5 (4)	1.65 ± 0.4 (7)
Luteinizing hormone (ng/ml)	11.4 ± 0.7 (4)	11.9 ± 1.1 (7)

### Contribution of pregnancy and postpartum anhedonia to inadequate maternal behaviour in CAST KO

**Figure 5:**
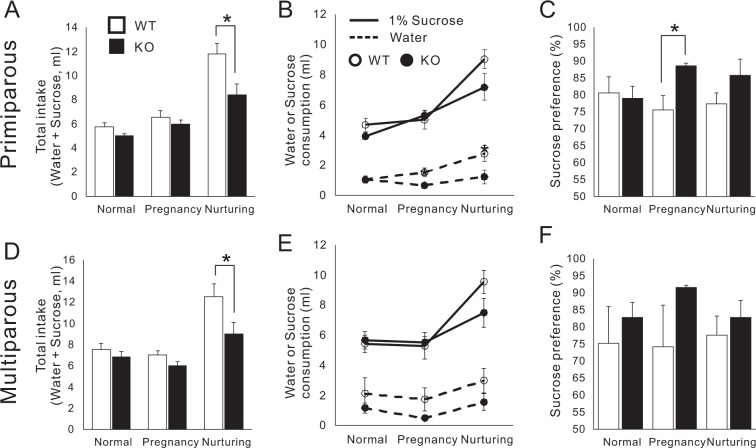
CAST KO mice exhibited less water intake during nurturing and higher sucrose preference during primiparous pregnancy. **(A–C)** The sucrose preference test was performed with WT and KO females before and after their first pregnancies. Total water intake increased during nurturing in both WT and KO mice, however the amount was significantly lower in KO mice (**A**). While both WT and KO mice preferred to drink the sucrose water (**B**), and sucrose preference was significantly higher in KO mice during their primiparous pregnancy (**C**). **(D–F)** The same test was performed on multiparous females. Although sucrose preference was higher in the KO mice during pregnancy, the difference was not significant (**F**). WT (10), KO (9), mean ± SEM, *p < 0.05 (Student’s *t*-test).

Because symptoms of depression- and anxiety-like behaviours during postpartum periods cause abnormal pup care^13^, we used the sucrose test to assess WT and KO mice during pregnancy and nurturing. First of all, total intake (water and sucrose water) was analyzed before and after pregnancy and nurturing (Fig. 5A,D). Before pregnancy, the total intake did not differ significantly between WT and KO mice. During nurturing, the total intake increased in both WT and KO mice (primiparous and multiparous WT: nurturing vs. normal and pregnancy, **p < 0.01; primiparous KO: nurturing vs. normal, **p < 0.01; nurturing vs. pregnancy, *p < 0.05; multiparous KO: nurturing vs. pregnancy, *p < 0.05; one-way ANOVA, Tukey’s test), however, the intake was significantly lower in the KO mice (Fig. 5A,D). Next, the consumption of each water and sucrose water and the sucrose preference were investigated (Fig. 5B,C,E,F). Although lower sucrose preference on this test indicates core symptoms of depression and anhedonia^34,35^, both WT and KO mice showed preference for sucrose, and this preference was significantly higher in KO mice pregnant for the first time (Fig. 5C). The altered motivation to consume sucrose in primiparous KO mice might have affected their postpartum maternal behaviours. In the second time pregnant, sucrose preference during pregnancy was slightly higher in the KO mice, but not significantly different from that in the WT mice (Fig. 5F). These results paralleled the recovered crouching time and weaning rate in multiparous KO mice.

## Discussion

Maternal neglect greatly reduces the survival of offspring and is now a prevalent issue in many countries. Initial sociological studies revealed how child neglect influences the development of children physically, mentally, and socially, yet the roots of neglect from the mother’s side has not been thoroughly investigated. Maternal nurturing comprises complex and widely varying behaviours, such as nursing and hovering over pups (care), retrieving pups to the nest (motivation), and protecting pups from enemies (aggression)^2^. Here, we show that deletion mutants for CAST (a presynaptic release machinery protein) have the propensity to neglect pups during primiparous maternal care, as evidenced by impaired nest making and hyperactivity during nurturing (Figs. 1, 2). Moreover, although virgin CAST KO females showed interest in pups, they cared less for the pups (Fig. 3), indicating that CAST KO females could not properly switch to maternal behaviour.

Maternal behaviours are governed by hormonal changes during pregnancy and sensory cues received from pups, both of which modulate aspects of neurotransmitter release^2,11,36^. CAST is a CAZ protein that is known to assist in forming the presynaptic neurotransmitter-release site^15–17,37,38^. Immunoblotting of tissue homogenates indicated limited expression of CAST in the brain, while the broad expression of ELKS (CAST2) included the brain, spleen, lung, liver, testis, and islet^17,23,39^. The Allen Brain Atlas characterizes CAST and ELKS mRNA in the brain, and has evaluated the broad expression of CAST, with expression values >20 in the hippocampus and cortex, >10 in the thalamus and hypothalamus, and >5 in the cerebellum. In contrast, ELKS expression is more limited in the cerebellum, with a value of about 3 (Allen Brain Atlas, https://mouse.brain-map.org). CAST KO mice are basically viable and fertile with impaired management of exploratory drive and visual processing in the retina^24,26^. We found that while postpartum CAST KO dams were hyperactive, the degree to which male and virgin female CAST KO mice explored novel environments was comparable to that of WT controls (Fig. 2C,D). In a previous report, highly active dams also neglected their pups, including a high rate of cannibalization^40^. Here, CAST KO have exhibited hyperactivity during postpartum periods, which can be the cause of neglect (as evidenced by less crouching time), but we do not yet know which neural circuits are responsible.

Because olfactory cues have an important role in maternal behaviour, we next investigated olfaction ability in WT and KO virgin females to determine whether the observed behaviour was related to olfaction (Fig. 2E–H). The CAST KO mice were able to detect and habituate to odorants, and their ability to discriminate the difference between male and female urinary odours was as good as that in the WT mice. Therefore, olfactory cues from pups are likely successfully received, as evidenced by the comparable contact time to pups between WT and KO dams and virgin female mice (Fig. 3). However, because CAST is expressed in the olfactory bulb and olfactory cortex (Allen Brain Atlas), we cannot yet rule out the possibility that impaired transmission of olfactory information is related to the observed maternal behaviour.

Here, we first expected that neglect in CAST KO dams might be caused by impaired OXT release. Indeed, previous studies indicated an essential role for CAST in recruiting Ca^2+^ channels that can trigger neurotransmitter release at the active zone^21,22,25,41–43^, as well as the OXT release depends on Ca^2+^ influx into the axon terminal^44,45^. Our findings showed that magnocellular neuron terminals in the posterior pituitary were intensely labelled for CAST and OXT (Fig. 4A), though plasma OXT concentration did not differ between WT and KO mice (Fig. 4C). This was likely because CAST family protein ELKS, which also regulates the Ca^2+^-triggered exocytosis at pancreatic β cells^23,46^, could facilitate OXT release from the posterior pituitary in CAST KO mice (Fig. 4A). Normal OXT release to the peripheral system was confirmed by the proper development of mammary glands in CAST KO mice (Fig. 1). In addition to peripheral OXT release, hypothalamic OXT neurons release OXT as a neurotransmitter in the brain. Most maternal behaviour is regulated by this source of OXT^8,11,47^. In the case of CD38 KO mice that exhibit deficits in maternal behaviour, plasma OXT was low, and tissue OXT was high, indicating secretion was selectively and severely impaired^8^. Therefore, we speculate that because the family protein ELKS would compensate for the OXT release from the terminal of hypothalamic neurons in the pituitary of CAST KO mice, OXT secretion into the brain could also be regulated by other active zone proteins including ELKS. Here, we would like to note that in contrast to the essential roles of OXT and its receptor^6^, OXT receptor conditional KO in the forebrain showed potentially normal maternal behaviour^48^. Furthermore, studies show that neurotransmitters including dopamine, serotonin, and norepinephrine are altered in the postpartum mouse brain^2,36^, which indicates that several factors aside from OXT facilitate maternal behaviour. Therefore, brain regions associated with maternal behaviour and the precise correlation between specific neurotransmitter levels within specific circuits and deficient maternal behaviour in CAST KO dams remains to be determined by future investigations.

The results of the sucrose preference test (Fig. 5) suggest that CAST KO dams are susceptible to pregnancy and postpartum mood disorder, which increases the risk for pup neglect. First, we found that the total intake during nurturing was higher in WT and KO mice, but the amount was significantly lower in CAST KO dams. Water consumption is regulated by peripherally released vasopressin and OXT from pituitary, but the serum OXT levels did not significantly differ between WT and KO mice (Fig. 4D). Since the role of CAST in the presynaptic release machinery at the hippocampal synapse indicated a reduced release probability^22^, CAST can contribute precise regulation of hormonal release, which cannot be determined by looking at serum OXT levels. Second, we would like to note the increase in sucrose preference at pregnancy in CAST KO mice (Fig. 5C). The reduction of sucrose preference is used to detect anhedonia^34^, and pregnancy-related depression was indicated in a study of GABA_A_ receptor KO dams in which the preference was significantly reduced^13^. In contrast, an increase in preference was indicated depression-like behaviour with a mouse model for schizophrenia^35^. Therefore, the change of preference in CAST KO dams would predict modification of feeling caused by pregnancies. Recent clinical research has shown that corticotropin-releasing factor (CRF) is a key stress peptide that facilitates anti-maternal neuromodulation^9^. Similar to OXT, CRF is released peripherally from the pituitary as well as centrally in cortical and subcortical areas including the medial preoptic area (MPOA) and amygdala, which are well-known brain regions related to maternal care and emotions, respectively. Additionally, the motivation for maternal care induced by the dopaminergic system could also have essential roles^2^.

In this study, we have suggested that the modulation of release machinery is one of the key factors that motivates maternal behaviour. Abnormal maternal behaviour resulting from defects in release machinery proteins has been reported previously in deletion mutants for Rim (a CAST binding protein) and the Rim effector protein Rab3 (the GTP-binding protein of synaptic vesicles). These mutant dams did not take care of their litters even after multiple pregnancies^27,49,50^. However, complex maternal behaviour is regulated by several neurotransmitters in numerous brain regions, which implies that the neglect observed in CAST KO dams likely results from comprehensive effects regulated by multiple neuromodulators. Therefore, specifying the primary factors responsible for these deficits in maternal behaviours is difficult. Nevertheless, the higher weaning rate in multiparous dams shows that the neglect seen in CAST KO dams could be restored by experience. However, the crouching time for multiparous dams during nurturing (Fig. 1J), and sucrose preference at pregnancy (Fig. 5F) indicated that although not significant, the mice still had a tendency toward the behaviours exhibited by primiparous KO dams and their maternal behaviours were only partially recovered. This modification of neuronal transmission has a few possible explanations. First, the responsible portion of the neuronal network is plastically changed by experience, allowing dams to take care of litters. Second, while the principal network remains impaired due to CAST KO, compensatory remodelling of another network can recover some maternal behaviour. Future research using CAST KO dams and identifying brain regions and/or circuits responsible for CAST-dependent maternal behaviour should provide new insights into the presynaptic neural mechanisms of pup neglect, which could help us better understand the social problem of maternal neglect in humans.

## Materials and Methods

### Mice

The CAST KO strain was bred from heterozygotes (HT) and genotyped as previously described^26^. The use of animals was approved by the Institutional Committee for the Care and Use of Experimental Animals at the University of Yamanashi (protocol # A25-33, A30-21). All experiments were conducted according to the recommendations in the *Guidelines for Proper Conduct of Animal Experiments* of the Science Council of Japan (2006). All efforts were made to reduce the number and suffering of animals used. For the maternal behaviour analysis, the siblings of WT and KO females from the HT breeding pair were crossed with WT males. Therefore, pups were all WT from WT dams or HT from KO dams. Adult mice used in this study were 9–12 weeks old unless otherwise indicated and all pups were 0–3 days old.

### Nurturing analysis of postpartum female mice

To test whether maternal behaviours were impaired in CAST KO females, nurturing analyses were assayed as described previously, with some modification^8,51^. All females were individually housed once they became pregnant and were monitored for pup birth. The retrieval assay was performed in the female’s home cage using the following procedure. After recognition of newborn pups, each new mother was monitored for 20 min with minimal disruption. Next, all of her pups were removed and kept warm for 1 h in a separate cage. Subsequently, the mother was briefly removed from her home cage and a pup was placed in each corner of the cage (except the corner with the nest; n = 3 pups). The mother was then returned to her nest facing the wall. Nurturing behaviour was monitored continuously over the next 20 min and the following data points were recorded: latency to retrieve each pup, the total number of pups retrieved, and the time spent crouching over all three pups in the nest. Retrieval was defined as the mother picking up a pup in her mouth and transporting it to the nest. If she picked up and dropped the same pup more than once en route to the nest, the retrieval was not scored until the pup was in the nest. Crouching was defined as the mother arching her back and assuming the nursing posture with all 3 pups under her ventral surface in the nest, although the pups did not actually have to be nursing. The ambulation of mice for 5–15 min at the initial monitoring and during nurturing was analysed from video images captured with ImageOF (freely available from Mouse Phenotype Database, http://www.mouse-phenotype.org/).

### Nesting score

Individually housed pregnant females were provided nesting material a day before testing. Nest quality was evaluated following methods in a previous report^52^. The score (1–5) was determined by how the material had been manipulated by the mice. If the material was untouched, the score was 0. If the material had been moved to the nest site, but remained in its original form, the score was 1. We have never observed material spread throughout the cage. A proper nest is a hollowed-out region surrounded by a bank. If attempts had been made to shape the material into a nest, the nest shape was considered. If the nest was flat with no shallow walls, the score was 2. If it had a slightly cupped shape in which the wall of the nest was less than half the height of a dome that would cover a mouse, the score was 3. If the wall was half the height of a dome, the score was 4. If the wall was taller than half the height of a dome, which may or may not fully enclose the nest, the score was 5. The nest was further confirmed 3 days after and evaluated.

### Maternal behaviour of virgin female mice

To quantify parental responsiveness, the nurturing analysis was performed using virgin females as described previously^53^. All subject animals were exposed to pups for the first time in their life, aside from their own littermates. Adult virgin females (10–16 wk) were individually housed for at least 1 d before the experiment. On the test day, each animal was exposed to more than 1 h starved three of P1–5-day BL6 pups. One pup was placed in each corner of the cage distant from the nest, and the virgin mouse was returned to her nest facing the wall. The cage was continually observed for the next 30 min, and the following data points were recorded: latency to sniff a pup for the first time, latency to retrieve each pup to the nest, and time spent crouching over all three pups. If any of the pups was attacked during the test, all pups were immediately removed. The assay was repeated on two sequential days.

### Sucrose preference test

The sucrose preference test was performed as previously described^35^. Mice were individually housed before the experiment. Animals were given a water bottle containing water and a second bottle containing 1% sucrose (with the left/right location counterbalanced across animals) for three consecutive days. Both bottles were removed each day and weighed. Sucrose preference was calculated according to the formula: % preference = (Δ weight sucrose)/(Δ weight sucrose + Δ weight water) × 100.

### Open field test

The open-field test was conducted as previously described^54^. Briefly, the test was conducted using a circular apparatus with grey walls (diameter, 80 cm; height, 45 cm). The mice were allowed to freely explore the environment for 30 min and ambulation was recorded and analysed with a video-computerized tracking system (SMART, Panlab SL). For the analysis, the field was divided into two concentric circles with an inner region that was 60 cm in diameter.

### Olfactory habituation and discrimination test

The olfactory test was performed as described previously, with some modifications^29,30^. Briefly, mice were individually housed more than a week and handled for 2 min, twice a day prior to the test. On the test day, mice were pretrained with mineral oil-laced cotton swabs for three presentations (60 s, 2 min intervals), and then exposed sequentially to two odorants (100 µM 1-octanol (25506-62, Nacalai Tesque) and 100 µM Benzaldehyde (04006-62, Nacalai Tesque)) with three presentations for each odorant with the same mineral oil protocol. On a separate day, the mice performed a discrimination test in the same way as the odorants using male and female urinary odours, which were collected and pooled from a different group of 15 male and 17 female mice prior to the test. Cumulative time spent sniffing the tip during 1 min trial was scored off-line from the video recording. Sniffing was distinguished when the mouse was oriented towards the cotton tip with its nose 1 cm or closer to the tip.

### Immunohistochemistry

Under deep pentobarbital anaesthesia, mice were transcardially fixed with 4% paraformaldehyde and 10% picric acid in 0.1 M phosphate buffer (pH 7.4). Pituitary glands were dissected and sectioned at a thickness of 10 µm on a cryostat (HM525 NX, Thermo). The collected sections were blocked for 1 hour in blocking solution (2% normal horse serum, 10% block ace [Dainippon Pharmaceutical], 0.2% Triton X-100 in PBS) and incubated with primary antibodies in blocking solution overnight. The following primary antibodies were used: anti-oxytocin, anti-growth hormone, anti-prolactin, and anti-thyroid stimulating hormone (all antibodies were made from rabbit, kindly provided by Gunma University). For immunohistochemical analysis of CAST and ELKS, we performed ethanol and acetone fixes as described previously^55^. In brief, freshly frozen pituitaries were sectioned at a thickness of 10 µm on the cryostat, and fixed in 95% ethanol at 4 °C for 30 min and in acetone at room temperature for 1 min. After blocking, sections were incubated with anti-CAST (1/100, guinea pig or rabbit)^17,33^ and anti-ELKS (1/100, rabbit)^39^ antibodies. The sections were further processed with appropriate Alexa Fluor-conjugated secondary antibodies for 1 h at room temperature. Immunolabelled samples were imaged using a confocal laser microscope (FV1200, Olympus).

### OXT extraction and ELISA assay of hormones

Collected plasma was stored at −80 °C until assay. Plasma samples were first extracted using C18 reversed-phase resin (C18 Spin Column, Pierce) to eliminate the effect of potentially interfering molecules. OXT was eluted with 70% acetonitrile, and then the solvent was evaporated. Quantitative determination of OXT was performed with a competitive immunoassay ELISA kit (Enzo) following the manufacturers’ protocols and detected using a microplate reader (SpectraMax 340, Molecular Devices). All samples were run in duplicate and within the same assay. The intra-assay coefficient of variation was 13.7% for standards, and 20.7% for samples. Values of growth hormone and luteinizing hormone in collected serum were examined by clinical test services (LSI Medience Corporation, Mitsubishi Chemical Holdings Group, Japan).

### Statistical analysis

Data are presented as mean ± SEM. Groups were compared using 2-tailed Student’s t-tests or one-way ANOVA with Tukey’s post hoc test. *p* < 0.05 was considered statistically significant throughout the study.
